# Supplementary material for: Modeling glioblastoma heterogeneity as a dynamic network of cell states
Source: Mol Syst Biol. 2021 Sep 16;17(9):e10105. doi: 10.15252/msb.202010105 (PMC8444284; doi:10.15252/msb.202010105)
Supplement: Supplementary file 5 — Source Data for Figure 3 [file MSB-17-e10105-s001.zip › Figure3A_sourcedata/GSEA_3065/hallmarks_state6.GseaPreranked.1623416308702/HALLMARK_MTORC1_SIGNALING.html]

Details for gene set HALLMARK\_MTORC1\_SIGNALING[GSEA]

|  || Dataset | state6 |
| Phenotype | NoPhenotypeAvailable |
| Upregulated in class | na\_neg |
| GeneSet | HALLMARK\_MTORC1\_SIGNALING |
| Enrichment Score (ES) | -0.3925531 |
| Normalized Enrichment Score (NES) | -1.6856216 |
| Nominal p-value | 0.0 |
| FDR q-value | 0.010391379 |
| FWER p-Value | 0.08 |
Table: GSEA Results Summary

  

Fig 1: Enrichment plot: HALLMARK\_MTORC1\_SIGNALING      
 Profile of the Running ES Score & Positions of GeneSet Members on the Rank Ordered List

  

| PROBE | GENE SYMBOL | GENE\_TITLE | RANK IN GENE LIST | RANK METRIC SCORE | RUNNING ES | CORE ENRICHMENT || 1 | CDKN1A |  |  | 1 | 0.477 | 0.0318 | No |
| 2 | FDXR |  |  | 56 | 0.241 | 0.0417 | No |
| 3 | BTG2 |  |  | 63 | 0.234 | 0.0567 | No |
| 4 | DDIT3 |  |  | 205 | 0.175 | 0.0520 | No |
| 5 | NUFIP1 |  |  | 302 | 0.151 | 0.0510 | No |
| 6 | CCNG1 |  |  | 319 | 0.148 | 0.0591 | No |
| 7 | EIF2S2 |  |  | 347 | 0.144 | 0.0656 | No |
| 8 | DDIT4 |  |  | 357 | 0.142 | 0.0741 | No |
| 9 | PNO1 |  |  | 440 | 0.130 | 0.0733 | No |
| 10 | SYTL2 |  |  | 539 | 0.119 | 0.0699 | No |
| 11 | TCEA1 |  |  | 576 | 0.116 | 0.0735 | No |
| 12 | PSPH |  |  | 603 | 0.113 | 0.0781 | No |
| 13 | SQSTM1 |  |  | 719 | 0.104 | 0.0717 | No |
| 14 | SLC1A4 |  |  | 741 | 0.103 | 0.0761 | No |
| 15 | BCAT1 |  |  | 759 | 0.102 | 0.0810 | No |
| 16 | RAB1A |  |  | 811 | 0.097 | 0.0816 | No |
| 17 | ACTR2 |  |  | 833 | 0.096 | 0.0856 | No |
| 18 | SHMT2 |  |  | 843 | 0.095 | 0.0909 | No |
| 19 | ASNS |  |  | 850 | 0.094 | 0.0965 | No |
| 20 | SRD5A1 |  |  | 867 | 0.093 | 0.1009 | No |
| 21 | MTHFD2 |  |  | 926 | 0.090 | 0.1002 | No |
| 22 | PPP1R15A |  |  | 968 | 0.087 | 0.1013 | No |
| 23 | GLRX |  |  | 1028 | 0.084 | 0.1001 | No |
| 24 | STC1 |  |  | 1133 | 0.079 | 0.0933 | No |
| 25 | NFKBIB |  |  | 1179 | 0.076 | 0.0932 | No |
| 26 | PGM1 |  |  | 1302 | 0.071 | 0.0838 | No |
| 27 | YKT6 |  |  | 1307 | 0.071 | 0.0881 | No |
| 28 | PIK3R3 |  |  | 1321 | 0.070 | 0.0913 | No |
| 29 | GSK3B |  |  | 1382 | 0.068 | 0.0889 | No |
| 30 | QDPR |  |  | 1420 | 0.067 | 0.0891 | No |
| 31 | ARPC5L |  |  | 1538 | 0.063 | 0.0797 | No |
| 32 | ACTR3 |  |  | 1668 | 0.058 | 0.0687 | No |
| 33 | HSPE1 |  |  | 1803 | 0.054 | 0.0567 | No |
| 34 | BHLHE40 |  |  | 1880 | 0.051 | 0.0513 | No |
| 35 | SERP1 |  |  | 1912 | 0.050 | 0.0511 | No |
| 36 | PDAP1 |  |  | 1999 | 0.048 | 0.0443 | No |
| 37 | ATP6V1D |  |  | 2082 | 0.045 | 0.0379 | No |
| 38 | SLC2A3 |  |  | 2103 | 0.045 | 0.0385 | No |
| 39 | GTF2H1 |  |  | 2146 | 0.043 | 0.0366 | No |
| 40 | STIP1 |  |  | 2150 | 0.043 | 0.0391 | No |
| 41 | GPI |  |  | 2151 | 0.043 | 0.0420 | No |
| 42 | PSMG1 |  |  | 2229 | 0.040 | 0.0358 | No |
| 43 | MLLT11 |  |  | 2335 | 0.038 | 0.0261 | No |
| 44 | POLR3G |  |  | 2381 | 0.036 | 0.0234 | No |
| 45 | RIT1 |  |  | 2457 | 0.035 | 0.0170 | No |
| 46 | IDH1 |  |  | 2508 | 0.033 | 0.0134 | No |
| 47 | RPA1 |  |  | 2511 | 0.033 | 0.0154 | No |
| 48 | GBE1 |  |  | 2517 | 0.033 | 0.0170 | No |
| 49 | PSAT1 |  |  | 2600 | 0.031 | 0.0095 | No |
| 50 | COPS5 |  |  | 2629 | 0.030 | 0.0083 | No |
| 51 | CDC25A |  |  | 2768 | 0.027 | -0.0058 | No |
| 52 | TBK1 |  |  | 2840 | 0.026 | -0.0123 | No |
| 53 | PSMD14 |  |  | 2855 | 0.025 | -0.0123 | No |
| 54 | HSPA4 |  |  | 2968 | 0.023 | -0.0238 | No |
| 55 | UBE2D3 |  |  | 3038 | 0.021 | -0.0303 | No |
| 56 | PLK1 |  |  | 3116 | 0.020 | -0.0380 | No |
| 57 | CYB5B |  |  | 3128 | 0.019 | -0.0379 | No |
| 58 | PPA1 |  |  | 3215 | 0.017 | -0.0467 | No |
| 59 | VLDLR |  |  | 3225 | 0.017 | -0.0466 | No |
| 60 | HMBS |  |  | 3229 | 0.017 | -0.0459 | No |
| 61 | TXNRD1 |  |  | 3380 | 0.014 | -0.0623 | No |
| 62 | GSR |  |  | 3436 | 0.012 | -0.0679 | No |
| 63 | PSME3 |  |  | 3470 | 0.012 | -0.0709 | No |
| 64 | PPIA |  |  | 3964 | 0.001 | -0.1280 | No |
| 65 | PGK1 |  |  | 3973 | 0.001 | -0.1289 | No |
| 66 | ALDOA |  |  | 4006 | 0.001 | -0.1325 | No |
| 67 | ACACA |  |  | 4189 | -0.003 | -0.1534 | No |
| 68 | NMT1 |  |  | 4270 | -0.004 | -0.1624 | No |
| 69 | XBP1 |  |  | 4331 | -0.006 | -0.1690 | No |
| 70 | PFKL |  |  | 4347 | -0.006 | -0.1703 | No |
| 71 | CCT6A |  |  | 4362 | -0.006 | -0.1716 | No |
| 72 | UFM1 |  |  | 4474 | -0.008 | -0.1839 | No |
| 73 | SLC6A6 |  |  | 4739 | -0.013 | -0.2136 | No |
| 74 | DDX39A |  |  | 4809 | -0.014 | -0.2206 | No |
| 75 | ACLY |  |  | 4883 | -0.016 | -0.2280 | No |
| 76 | SLC7A5 |  |  | 4959 | -0.017 | -0.2355 | No |
| 77 | LGMN |  |  | 4983 | -0.018 | -0.2370 | No |
| 78 | EEF1E1 |  |  | 5148 | -0.021 | -0.2546 | No |
| 79 | SKAP2 |  |  | 5223 | -0.023 | -0.2617 | No |
| 80 | NFYC |  |  | 5250 | -0.023 | -0.2631 | No |
| 81 | RDH11 |  |  | 5274 | -0.024 | -0.2642 | No |
| 82 | SLC7A11 |  |  | 5291 | -0.024 | -0.2644 | No |
| 83 | PSMD13 |  |  | 5303 | -0.024 | -0.2641 | No |
| 84 | MTHFD2L |  |  | 5314 | -0.025 | -0.2636 | No |
| 85 | BUB1 |  |  | 5377 | -0.026 | -0.2691 | No |
| 86 | RRP9 |  |  | 5380 | -0.026 | -0.2676 | No |
| 87 | G6PD |  |  | 5387 | -0.026 | -0.2665 | No |
| 88 | HMGCS1 |  |  | 5446 | -0.027 | -0.2714 | No |
| 89 | PRDX1 |  |  | 5508 | -0.029 | -0.2766 | No |
| 90 | ADIPOR2 |  |  | 5514 | -0.029 | -0.2752 | No |
| 91 | ERO1A |  |  | 5668 | -0.032 | -0.2908 | No |
| 92 | HPRT1 |  |  | 5723 | -0.033 | -0.2949 | No |
| 93 | PITPNB |  |  | 5784 | -0.034 | -0.2995 | No |
| 94 | GOT1 |  |  | 5954 | -0.037 | -0.3167 | No |
| 95 | TM7SF2 |  |  | 6044 | -0.039 | -0.3244 | No |
| 96 | GMPS |  |  | 6067 | -0.040 | -0.3243 | No |
| 97 | TUBG1 |  |  | 6104 | -0.040 | -0.3257 | No |
| 98 | PSMB5 |  |  | 6157 | -0.042 | -0.3290 | No |
| 99 | SC5D |  |  | 6168 | -0.042 | -0.3273 | No |
| 100 | LTA4H |  |  | 6318 | -0.045 | -0.3416 | No |
| 101 | FADS2 |  |  | 6359 | -0.046 | -0.3432 | No |
| 102 | GGA2 |  |  | 6361 | -0.046 | -0.3402 | No |
| 103 | ATP5MC1 |  |  | 6395 | -0.047 | -0.3409 | No |
| 104 | TFRC |  |  | 6511 | -0.050 | -0.3508 | No |
| 105 | PSMC6 |  |  | 6525 | -0.050 | -0.3490 | No |
| 106 | PHGDH |  |  | 6659 | -0.054 | -0.3608 | No |
| 107 | ETF1 |  |  | 6699 | -0.056 | -0.3615 | No |
| 108 | SLC37A4 |  |  | 6732 | -0.057 | -0.3615 | No |
| 109 | ADD3 |  |  | 6818 | -0.059 | -0.3673 | No |
| 110 | M6PR |  |  | 6966 | -0.064 | -0.3801 | No |
| 111 | MAP2K3 |  |  | 6986 | -0.064 | -0.3780 | No |
| 112 | IGFBP5 |  |  | 7011 | -0.065 | -0.3764 | No |
| 113 | ELOVL5 |  |  | 7151 | -0.069 | -0.3879 | Yes |
| 114 | IFRD1 |  |  | 7168 | -0.070 | -0.3851 | Yes |
| 115 | NAMPT |  |  | 7188 | -0.071 | -0.3826 | Yes |
| 116 | CXCR4 |  |  | 7242 | -0.072 | -0.3839 | Yes |
| 117 | CCNF |  |  | 7245 | -0.073 | -0.3792 | Yes |
| 118 | UNG |  |  | 7249 | -0.073 | -0.3747 | Yes |
| 119 | PSMC4 |  |  | 7254 | -0.073 | -0.3703 | Yes |
| 120 | UCHL5 |  |  | 7369 | -0.077 | -0.3784 | Yes |
| 121 | IMMT |  |  | 7374 | -0.077 | -0.3737 | Yes |
| 122 | HSPD1 |  |  | 7433 | -0.079 | -0.3751 | Yes |
| 123 | ELOVL6 |  |  | 7498 | -0.081 | -0.3771 | Yes |
| 124 | CYP51A1 |  |  | 7511 | -0.082 | -0.3730 | Yes |
| 125 | ENO1 |  |  | 7559 | -0.084 | -0.3728 | Yes |
| 126 | EBP |  |  | 7564 | -0.084 | -0.3676 | Yes |
| 127 | SSR1 |  |  | 7569 | -0.085 | -0.3624 | Yes |
| 128 | LDLR |  |  | 7648 | -0.088 | -0.3656 | Yes |
| 129 | P4HA1 |  |  | 7667 | -0.089 | -0.3617 | Yes |
| 130 | TOMM40 |  |  | 7683 | -0.090 | -0.3574 | Yes |
| 131 | AURKA |  |  | 7700 | -0.091 | -0.3532 | Yes |
| 132 | PLOD2 |  |  | 7718 | -0.092 | -0.3490 | Yes |
| 133 | INSIG1 |  |  | 7721 | -0.092 | -0.3431 | Yes |
| 134 | TPI1 |  |  | 7757 | -0.093 | -0.3409 | Yes |
| 135 | EDEM1 |  |  | 7798 | -0.096 | -0.3391 | Yes |
| 136 | DHCR7 |  |  | 7806 | -0.096 | -0.3335 | Yes |
| 137 | IDI1 |  |  | 7851 | -0.099 | -0.3320 | Yes |
| 138 | PSMA3 |  |  | 7871 | -0.100 | -0.3275 | Yes |
| 139 | MCM4 |  |  | 7900 | -0.101 | -0.3240 | Yes |
| 140 | SCD |  |  | 7908 | -0.102 | -0.3180 | Yes |
| 141 | USO1 |  |  | 7963 | -0.105 | -0.3173 | Yes |
| 142 | PSMA4 |  |  | 7995 | -0.107 | -0.3137 | Yes |
| 143 | RRM2 |  |  | 8001 | -0.107 | -0.3071 | Yes |
| 144 | CACYBP |  |  | 8012 | -0.108 | -0.3011 | Yes |
| 145 | TMEM97 |  |  | 8049 | -0.110 | -0.2979 | Yes |
| 146 | HSPA9 |  |  | 8097 | -0.113 | -0.2957 | Yes |
| 147 | MCM2 |  |  | 8099 | -0.114 | -0.2882 | Yes |
| 148 | FADS1 |  |  | 8186 | -0.120 | -0.2901 | Yes |
| 149 | SDF2L1 |  |  | 8235 | -0.124 | -0.2874 | Yes |
| 150 | LDHA |  |  | 8261 | -0.127 | -0.2818 | Yes |
| 151 | ACSL3 |  |  | 8295 | -0.132 | -0.2768 | Yes |
| 152 | DHCR24 |  |  | 8321 | -0.134 | -0.2707 | Yes |
| 153 | GLA |  |  | 8383 | -0.144 | -0.2681 | Yes |
| 154 | NUP205 |  |  | 8386 | -0.145 | -0.2587 | Yes |
| 155 | PSMD12 |  |  | 8492 | -0.159 | -0.2602 | Yes |
| 156 | PSMC2 |  |  | 8501 | -0.161 | -0.2503 | Yes |
| 157 | SEC11A |  |  | 8542 | -0.171 | -0.2435 | Yes |
| 158 | SLC2A1 |  |  | 8552 | -0.173 | -0.2329 | Yes |
| 159 | FKBP2 |  |  | 8578 | -0.181 | -0.2237 | Yes |
| 160 | DHFR |  |  | 8590 | -0.186 | -0.2125 | Yes |
| 161 | ATP2A2 |  |  | 8624 | -0.200 | -0.2029 | Yes |
| 162 | SERPINH1 |  |  | 8646 | -0.212 | -0.1912 | Yes |
| 163 | CANX |  |  | 8735 | -0.270 | -0.1833 | Yes |
| 164 | RPN1 |  |  | 8736 | -0.272 | -0.1651 | Yes |
| 165 | HMGCR |  |  | 8759 | -0.301 | -0.1475 | Yes |
| 166 | CD9 |  |  | 8762 | -0.303 | -0.1274 | Yes |
| 167 | SQLE |  |  | 8767 | -0.319 | -0.1065 | Yes |
| 168 | CALR |  |  | 8773 | -0.330 | -0.0850 | Yes |
| 169 | HSPA5 |  |  | 8785 | -0.369 | -0.0615 | Yes |
| 170 | CTSC |  |  | 8791 | -0.448 | -0.0321 | Yes |
| 171 | HSP90B1 |  |  | 8794 | -0.487 | 0.0002 | Yes |
Table: GSEA details [plain text format]

  

Fig 2: HALLMARK\_MTORC1\_SIGNALING: Random ES distribution      
 Gene set null distribution of ES for **HALLMARK\_MTORC1\_SIGNALING**
